# Impact of the COVID-19 pandemic on cancer diagnosis, treatment and research in African health systems: a review of current evidence and contextual perspectives

**DOI:** 10.3332/ecancer.2021.1170

**Published:** 2021-01-14

**Authors:** Chukwudi A Nnaji, Jennifer Moodley

**Affiliations:** 1 Women’s Health Research Unit, School of Public Health and Family Medicine, Faculty of Health Sciences, University of Cape Town; Anzio Road, Observatory, 7925 Cape Town, South Africa; 2 Cancer Research Initiative, Faculty of Health Sciences, University of Cape Town, Anzio Road, Observatory, 7925 Cape Town, South Africa; 3 SAMRC Gynaecology Cancer Research Centre, University of Cape Town, 7925 Cape Town, South Africa

**Keywords:** COVID-19, pandemic, cancer care, oncology, access, social distancing, Africa

## Abstract

The coronavirus disease 2019 (COVID-19) pandemic has immensely disrupted health care services globally. The pandemic has been particularly disruptive for cancer services and more so in low-resource settings. In this narrative review, we highlight the reported impact of the COVID-19 pandemic on cancer prevention, screening, diagnosis, treatment and research across the African continent. We also explore ways in which identified structural and contextual constraints can be navigated for the re-escalation of oncological activities, while discussing how the pandemic has necessitated the reimagination of how oncology services can be delivered now and in the future. We conducted a literature search of MEDLINE (via PubMed) and Scopus for relevant articles and synthesised the findings thematically. In spite of the dearth of data, available evidence suggests a substantial impact of the pandemic on the various aspects of cancer management in African countries. Aggravating factors include pre-existing health system and cancer management gaps in many countries within the region, which are typically faced with inadequate availability of oncology resources, oncologists and other vital resources; in addition to the acute and lingering consequences of social distancing, movement restrictions and other public health measures implemented to contain the spread of the virus. As the pandemic evolves and movement restrictions are eased, there is a need for the timely and safe return to normal oncological care. This will require a risk-adjusted and multidisciplinary approach, with the aim of mitigating the further impact of the disruption on cancer patients, their families and healthcare providers.

## Introduction

The coronavirus disease 2019 (COVID-19) pandemic has immensely disrupted health care services globally [1]. The pandemic has been particularly disruptive for cancer services and more so in low-resource settings such as in many African countries and other low- and middle-income countries [2–5]. COVID-19 has impacted the landscape of cancer care in many ramifications, from prevention to screening and diagnosis, treatment, palliative care and patient follow-up, as well as logistics and supplies of cancer drugs and other essential commodities [6–8]. The disruptions have been attributed to a number of factors, including the redeployment of oncology healthcare workers to the COVID-19 frontline; inadequate supply of personal protective equipment (PPE) and shortage of anticancer drugs and other essential clinical supplies due to logistic constraints posed by movement restrictions and closure of country borders. Reduced demand for oncology services has also been attributed to the inability or unwillingness of cancer patients to visit health facilities due to movement restrictions or the heightened fear of COVID-19 [9, 10].

Furthermore, disruptions of cancer services have resulted from the considerations that cancer patients are a high-risk population for COVID-19 infection, severe disease and fatal outcomes, either due to the immunosuppression caused by the malignancy itself, or by anticancer treatments such as chemotherapy and radiotherapy [11, 12]. Consequently, reduction or suspension of in-person patient contacts with health facilities, delay of chemotherapy, hypo-fractionation of radiotherapy and rescheduling of cancer surgeries have been implemented in many oncology contexts [13, 14].

As of 5 October 2020, the African region had recorded 1,518,662 confirmed COVID-19 cases and 36,921 deaths [15]. The impact of the pandemic has been expected to be particularly dire in African contexts, where access to cancer services is typically suboptimal; with only a few countries having a functional national cancer-control plan, while out-of-pocket expenditure on cancer care remains highly prevalent [16, 17]. While the prevailing frailty of health systems and background challenges faced by the few available cancer care resources are expected to worsen the disruption of cancer services in the African region, the exact magnitude of impact remains uncertain. If left unmitigated, the difficulties in accessing oncology care posed by the pandemic can significantly aggravate existing cancer prevention, diagnostic and treatment gaps and can worsen the region’s overall cancer disease burden, morbidity and mortality in the long term. Hence, understanding the extent of the disruptions on the oncology landscape is important for planning and implementing the scale-up of oncology services to pre-pandemic levels. In this review, we highlight the reported impact on cancer prevention, diagnosis, treatment and research across the continent. We also explore ways in which identified structural and contextual constraints can be navigated for the re-escalation of oncological activities, while discussing ways in which the pandemic has necessitated the reimagination of how oncology services can be delivered now and beyond the pandemic.

To identify the relevant evidence on the the impact of the COVID-19 pandemic on the cancer control and management landscape in African contexts, we conducted a literature search of MEDLINE (via LitCOVID in PubMed) and Scopus. Reference lists of relevant articles were also scanned for potentially relevant articles. This review follows the evidence synthesis format of narrative reviews [18, 19]. Findings are summarised narratively and reported under the following themes:

## Cancer management recommendations in African settings

To mitigate the impact of the pandemic on cancer management, oncology centres and institutions across the continent have adopted clinical practice guidelines and recommendations to guide the re-organisation of cancer services to maximise the use of the limited available resources for cancer management in COVID-19 outbreak settings [3, 4, 20]. These measures were adapted based on international best practice recommendations and tailored to local contexts. Generally, it has been recommended that, whenever possible, cancer treatment should be delayed or suspended, as well as avoiding unnecessary hospitalisations to minimise the risk of patient exposure to COVID-19 and to free up health care resources for COVID-19 management [3, 21, 22]. Other common recommendations include optimal screening and testing of patients who attend cancer care facilities for COVID-19, triaging patients based on risk–benefit profiles and providing isolation rooms for patients presenting with symptoms of COVID-19 [21, 23, 24]. Considering the increased risk of severe infection associated with anticancer treatment such as chemotherapy, treatment modifications such as delays, de-escalation and interruptions have also been advised [3, 14, 20]. The use of existing digital health platforms has also been widely recommended to limit patients’ and oncology professionals’ physical interactions as a way of reducing the risk of COVID-19 infection transmission amongst cancer patients and oncologists [21, 25]. Shortening the length of stay of palliative care patients by providing home care, as well as limiting visitors from patients’ bedsides [9, 10, 25].

## Impact on cancer prevention services (including screening and vaccination)

While still limited, the evidence of the actual impact of the pandemic on African cancer screening and diagnostic programmes is beginning to emerge. In response to the early surge of COVID-19 cases, cancer screening programmes (including mammograms and pap smears) were suspended in Morocco [22, 26]. A similar situation has been reported In Uganda, where nation-wide COVID-19 movement restrictions and social distancing measures have led to the suspension of cancer screening services and awareness outreach programmes [27]. Many countries in Africa do not have organised screening programmes. The few that do focus mostly on cervical cancer screening, and even in those countries, screening coverage rates are often low, particularly in rural settings where coverage can be less than 10% of the target population [28]. Hence, the suspension of screening services will further worsen pre-existing limited access to cancer screening services.

On 26 March 2020, the World Health Organization (WHO) recommended the temporary suspension of mass vaccination campaigns and restriction of routine immunisation services in settings where community transmission of COVID-19 has been established [29]. These have led to significant reductions in immunisation encounters, including cancer-preventive vaccination against Hepatitis B Virus and Human papillomavirus (HPV), in 89% of countries in the WHO African region, as of June 2020 [30]. While HPV vaccination efforts have gained momentum in over the past decade, a majority of the countries in the African region lack national HPV immunisation programmes [28]. With only 11 countries having introduced HPV vaccination at the national level, the pandemic is capable of scuttling the plans of other countries intending to do so soon [31].

## Impact on early cancer diagnosis

A heightened perception of risk of contracting COVID-19 in a health care settings has led to the suspension of early diagnosis programmes globally [32, 33]. This impact is expected to be more remarkable in low-resource settings due to inadequate policies for infection control and resource constraints in the provision of PPE and delivery of cancer screening and diagnostic services. Delays in cancer diagnosis or treatment have the potential to worsen patient outcomes: including the increased likelihood of late diagnosis, metastatic disease, tumour progression from being curable to non-curable diseases; all of which affect patients’ overall survival [34–36]. Delays in scheduling diagnostic procedures have been reported in Ghana [3], as has the postponement of routine diagnostic imaging procedures in Nigeria [24]. While these reports are facility-level accounts and do not quantify the extent of the impact at the national level, it is reasonable to expect that these disruptions are likely to worsen the current trend of late diagnosis of cancer and with its attendant effect on cancer survival and treatment outcomes in those contexts.

## Impact on outpatient consultation

To ensure the safety of patients and oncologists, outpatient cancer consultations have been reorganised to reduce patients’ access to the hospital, with tele-medicine outpatient consultation approaches being increasingly considered [3, 37]. This has been the case in Tunisia, where the shutdown of public transport in addition to patients’ anxiety and unwillingness to attend their clinic appointments have led to a substantial decrease in the number of consultations: from 2,701 in May 2019 to 945 in the corresponding period in 2020 [38]. Similarly, a 40% decrease in the number of new outpatient cases compared to the same period of the year 2019 was observed in an oncology centre in Morocco [39]. There have also been reports of reduction in the frequency of outpatient consultation, while patients on follow-up and those who only require a prescription do not have to appear physically at the clinic or hospital in Nigeria, Ghana and Morocco [3, 10, 21].

## Impact on adult cancer treatment

Data on the impact of the pandemic on the various cancer therapeutic modalities within the African region remain sparse. However, there is significant evidence of how cancer treatment has been affected in some settings across the continent. Since the start of the current pandemic, oncologists have had to grapple with the dilemma between the clinical responsibility of maintaining optimal cancer treatment and care on the one hand; and the public health necessity of limiting the spread of the virus by reorganising oncological care to reduce or delay treatment visits, on the other hand [40]. Common recommendations have been to delay or postpone cancer treatment, avoid hospitalisation as much as possible to minimise the risk of patient exposure to COVID-19 and decrease the demand for health care services [3, 10, 23].

Movement restrictions, shortage of protective gear and insufficient onsite testing kits for patients and oncology staff have posed additional barrier to cancer treatment in African countries. This has been the case in Ghana, where oncology centres have had fewer number of patients and staff, while chemotherapy has been halted for some patients and the start of adjuvant therapies has been delayed for others [41]. Similarly, in Sudan, despite the low COVID-19 burden, cancer centres have established a contingency plan to defer new treatment referrals except for emergency cases, while suspending non-urgent intravenous chemotherapy and follow-up sessions [41].

The situation has not been any better for radiation therapy. While scheduled appointments for patients on radiotherapy are mostly maintained, many patients are unable to access treatment for various reasons [41]. It has been reported that cervical cancer patients in Zambia missed their course of radiotherapy within the recommended 8-week treatment period for the month of June 2020 [42]. This was attributed to the country’s inability to import essential supplies such as radioactive isotopes for High Dose Rate brachytherapy, following the nation-wide implementation of movement restrictions and border closures in response to the pandemic. Although scheduled radiotherapy has been sustained in oncology centres in Sudan, remote patients have faced difficulties in travelling to receive treatment [41]. In Ghana, reports indicate that only patients on concurrent chemo-radiotherapy will only receive radiotherapy, while new referrals, including emergencies, will be triaged on the basis of the effect of treatment delays on outcomes [41]. There is also some evidence that hypofractionated radiotherapy, which had limited acceptance prior to the COVID-19 era, has been adopted to reduce the frequency of hospital admissions [43].

For cancer surgical procedures, it was reported that access to cancer surgical services has been cancelled or reduced in 28.1% of hospitals surveyed in South Africa [44]. This follows earlier evidence from a modelling study which suggested that approximately 13,000 surgeries will be cancelled every week in the country, including many cancer surgeries [45]. The impact on palliative anticancer treatments has also been described. In Morocco, the recommendation has been to postpone anticancer palliative treatments for cancer patients diagnosed with COVID-19 until full recovery [21], and to halt second- and third-line palliative chemotherapy in Ghana [41].

## Impact on paediatric cancer care

The care of children with malignant solid tumours in sub-Saharan Africa is compromised by resource deficiencies that range from inadequate healthcare budgets and a paucity of appropriately trained personnel, to scarce laboratory facilities and inconsistent drug supplies. Patients face difficulties accessing healthcare, affording investigational and treatment protocols and attending follow-up [46, 47]. Children routinely present with advanced local and metastatic disease and many children cannot be offered any effective treatment [46]. While available evidence suggests that children are a low-risk population for COVID-19 susceptibility, and complications, the public health and social distancing measures that have been implemented to curb the spread of COVID-19 have had their collateral effect on the paediatric cancer diagnosis, treatment and care in many countries [48–51]. According to findings from a cross‐sectional survey of paediatric oncologists across 15 African countries including: Algeria, Benin, Burkina Faso, Central African Republic, Ivory Coast, Guinea, Gabon, Mali, Madagascar, Mauritania, Morocco, Democratic Republic of Congo, Senegal, Tunisia and Togo; the majority (54%) of the oncologists reported that COVID‐19 had a negative impact on the management of the six priority paediatric cancers of the WHO Global Initiative for Childhood Cancer in their centres. These negative impacts include the modifications of chemotherapy regimens due to drug shortages, delays of cancer surgeries , rescheduling of radiotherapy and blood supply shortages [9].

Another multi-country study involving some African countries, which assessed the barriers to paediatric oncology management during the COVID-19 pandemic, found that almost all oncology centres applied guidelines to optimise resource utilisation and patient safety, including delaying treatment visits, reducing staff and implementing social distancing. Overall, 30% of the participating centres reported that cancer care of children was negatively affected. Essential treatment, including chemotherapy, surgery and radiation therapy, was delayed in 29%–44% of centres, while 24% of centres restricted acceptance of new patients. Furthermore, about 70% of centres reported shortages in blood products, while 47%–62% reported interruptions in medication supplies, cancer surgery and radiation therapy [52].

## Impact on logistics and supply of cancer drugs and other essential medical commodities

With the current border closures, many African countries which rely on imported medical supplies have been faced with the acute challenge of supply shortages. There have been concerns of the high possibility of drug stock being depleted in Ghana, as almost all the systemic therapy medications used in the country are imported [3]. The situation has not been any better for radiation therapy, as many women have had to miss their course of radiotherapy in Zambia, as a result of the country’s inability to import essential cancer treatment supplies due to border closures [42]. In addition, it is not uncommon for oncology centres to delay or refuse the treatment of cancer patients due to shortage of COVID-19 test kits or insufficient availability of PPE [25, 41].

## Impact on cancer research

The disruption caused by the COVID-19 pandemic has not only affected the treatment and care of cancer patients, but has also undermined cancer research activities. This has been due the restrictions on non-essential services including closure of research sites and laboratories as well as the suspension of routine clinical research activities; the prioritisation of frontline COVID-19 care over research by clinicians and the re-allocation of health research resources to research on COVID-19 vaccine and therapeutics [53–55]. For instance, the recruitment of new research patients were suspended, while doctoral students and administrative staff were mostly advised to stay at home in a Moroccan context [21]. Overall, the amount of cancer specific research, publications and new clinical trials is expected to significantly decrease in the short- and long-term due to the pandemic [56].

## Plans for the re-escalation of cancer services and research activities

Reduced access to oncology services during the pandemic runs the very risk of increasing cancer morbidity and mortality, not only now but in the future [57]. Hence, it has become increasingly vital for the oncology community to plan for dealing with the backlog of cancer screening, diagnosis, treatment and research [6]. As movement restrictions are eased globally and across African countries, decision making around the timing and process of re-escalating cancer care will be challenging, particularly with reported incidents of ‘second waves’ and spikes in new COVID-19 cases already witnessed in an increasing number of countries [58, 59]. Scaling up oncology services to pre-pandemic levels will require a risk-adjusted, multidisciplinary and comprehensive measures to mitigate the further burden of the disruption on cancer patients, their families and healthcare providers. As more cancer care resources become available, a staged approach to the resumption of suspended services is a necessity.

The prevailing low COVID-19 testing capacity and insufficient supply of PPE in most African countries present an immediate challenge to the resumption of full oncology services and research. Inadequate testing capacity can place patients and healthcare personnel at a higher risk of infection, while undermining efforts to re-introduce previously restricted or suspended cancer services [37]. In spite of the resource constraints, there is best practice evidence that compliance with appropriate COVID-19 safety measures and practices in oncology care settings are feasible within African contexts, with the potential of reducing transmission and spread after the resumption of services [60]. Hence, it is imperative for health systems and oncology centres to ramp up the procurement of test kits, protective gears and other essential supplies to facilitate a time and safe re-escalation of oncology services.

Cancer programmes should continue offering cervical cancer screening and diagnostic services, following strict COVID-19 preventive measures. Preventive measures may include managing daily appointments to avoid overcrowding and maintaining physical dancing in queues. To catch up on missed screening opportunities, people visiting health care facilities for other health reasons could be offered age- and gender-appropriate cancer screening and early diagnostic services. While implementing the phased return of cancer treatment services, lingering movement restrictions may hinder patient referral to facilities offering the highest level of care at this period. Hence, facilities that may not have radiotherapy services should consider lower-level treatment options such as the use of chemotherapy, pending the feasibility of access to higher-level oncology centres [24]. It has also been recommended that regions should consider organising designated cancer treatment centres to start operating on a high volume of oncology cases. Furthermore, coordinated and dynamic monitoring systems have been recommended for monitoring the evolution of outstanding cancer screening, diagnosis and treatment cases. Such systems will help to facilitate the triage and transfer of patients to centres with the greatest capacity as demand for oncology services surge as movement restrictions are scaled down [61]. Since oncology care demands synergy between fields such as surgery, radiation oncology, medical oncology, radiology and pathology, institutions need to strengthen multi-disciplinary tumour board meetings. COVID-19 has further necessitated the need for joint decision making between the disciplines for each patient. This is because every situation needs to be assessed case by case [10].

Scaling up oncology services to pre-pandemic levels will require a multidisciplinary approach and increased communication between members of the oncology community for safely, efficiently and collaboratively returning to pre-pandemic standards of care [61]. Oncology care demands synergy between multiple specialties such as clinical oncology, haematology, surgery, radiation oncology, paediatrics, radiology and pathology and many others; institutions need to strengthen multidisciplinary tumour board interactions and joint decision making. This also calls for the engagement of less commonly engaged specialities such as psychology to deal with the psychosocial needs of cancer patients and their healthcare professionals in these unprecedented times.

Another important consideration for the re-escalation of cancer services is the continent’s large population of persons who are infected with human immunodeficiency virus (HIV), many of whom have cancer as a co-morbidity [62, 63]. Though it remains unclear how HIV infection will impact the management of cancer in the context of COVID-19, it may be prudent to take these patients into consideration when triaging and prioritising patient backlogs. Lastly, there is a need for ongoing monitoring and assessment of the impact of the pandemic on the oncology landscape in all ramifications and across various levels. This will help to generate time-sensitive and context-appropriate evidence for informing decision making for safely returning to pre-pandemic standards of care; as well as for dealing with future pandemics.

Some of the innovative approaches for adapting to the restrictive environment and mitigating the impact of the pandemic on cancer research include the adoption of remote practices to facilitate recruitment, patient contact, site visits, training and institutional review board communications. Other suggested approaches include the permission of protocol deviations, which may allow increased flexibility in the design and conduct of new trials [53]. Cancer researchers have also been urged to establish a clear road map to ensure a smooth and effective revival of cancer research immediately after the lockdown and movement restrictions have been eased, while stimulating investment in cancer research [56].

## COVID-19 and reimagining cancer care

The present COVID-19 pandemic period has, in an unprecedented way, created new opportunities for telemedicine innovations to remotely assist in the monitoring of patients in health management. There has been widespread adoption of telehealth visits, virtual check-in services, e-visits, remote care management and remote patient monitoring to sustain patient care and management and mitigate the impact of the pandemic [10, 37]. However, this strategy has technological and financial implications for both oncology centres and patients. It also requires stable Internet services, which may not be available in some communities particularly in rural areas [37]. Despite these challenges, the pandemic has shown that telemedicine can add value to oncology care in low-resource settings. Post COVID-19 consideration should be given to the use of telemedicine for patients for whom it is feasible and offers benefit, whilst continuing in-person consultations in situations where it is impractical. Research into the impact of this dual approach on inequity in access to care will be required.

The patient triage and treatment modification strategies necessitated by the COVID-19 pandemic have been unprecedented. These present opportunities for research to study the outcomes of patients who received modified and/or alternate cancer care schedules, to identify patient cohorts for whom treatment modifications may be beneficial in future pandemics.

There are also lessons to learn from the way in which COVID-19 related cancer research is advancing at a breakneck speed, as well as how tele-multidisciplinary tumour boards have had to improvise with virtual meetings, broadening collaboration across the globe, for challenging cancer cases as well as for research. Overall, the pandemic has allowed the rethinking of ways of improving health systems efficiency, flexibility and resilience [64].

## Conclusion

Evidence of the extent of the impact of the pandemic on cancer care in African countries is beginning to emerge. While the African continent is arguably the least affected in terms of the absolute number of confirmed COVID-19 cases and deaths, the real impact of the pandemic on cancer management and broader health services may not be as mild. The implications of these disruptions are grim in resource-limited settings, particularly in the African region, where cancer programmes are typically under-resourced. As the pandemic evolves and movement restrictions are eased, there is a need for the timely and safe restoration of oncological care and research.

## Conflicts of interest

None.

## Funding statement

None (the review was not funded).

## Authors’ contributions

The review was conceived by JM. CAN wrote the first manuscript. JM provided conceptual and analytical insights to the subsequent versions of the manuscript. Both authors read and approved the final manuscript.
